# Effects of Light-Emitting Diode Irradiation on Growth Characteristics and Regulation of Porphyrin Biosynthesis in Rice Seedlings

**DOI:** 10.3390/ijms18030641

**Published:** 2017-03-16

**Authors:** Lien Hong Tran, Sunyo Jung

**Affiliations:** School of Life Sciences and Biotechnology, BK21 Plus KNU Creative Bioresearch Group, Kyungpook National University, Daegu 41566, Korea; thlien91@yahoo.com

**Keywords:** growth, LED (light emitting diode), nuclear-encoded photosynthetic gene, photosynthetic pigment, porphyrin biosynthesis

## Abstract

We examined the effects of light quality on growth characteristics and porphyrin biosynthesis of rice seedlings grown under different wavelengths from light emitting diodes (LEDs). After 10 days of exposure to various wavelengths of LEDs, leaf area and shoot biomass were greater in seedlings grown under white and blue LEDs than those of green and red LEDs. Both green and red LED treatments drastically decreased levels of protoporphyrin IX (Proto IX) and Mg-porphyrins compared to those of white LED, while levels of Mg-Proto IX monomethyl ester and protochlorophyllide under blue LED were decreased by 21% and 49%, respectively. Transcript levels of *PPO1* were greatly upregulated in seedlings grown under red LED compared to white LED, whereas transcript levels of *HO2* and *CHLD* were upregulated under blue LED. Overall, most porphyrin biosynthetic genes in the Fe-porphyrin branch remained almost constant or upregulated, while most genes in the Mg-porphyrin branch were downregulated. Expression levels of nuclear-encoded photosynthetic genes *Lhcb* and *RbcS* noticeably decreased after exposure to blue and red LEDs, compared to white LED. Our study suggests that specific wavelengths of LED greatly influence characteristics of growth in plants partly through altering the metabolic regulation of the porphyrin biosynthetic pathway, and possibly contribute to affect retrograde signaling.

## 1. Introduction

**Figure 1:**
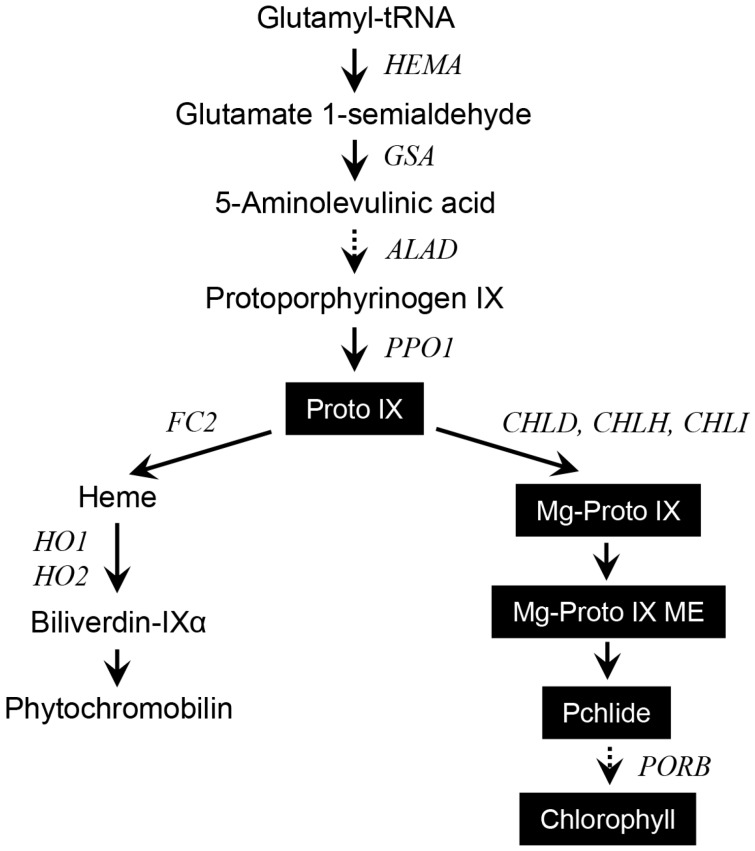
The porphyrin pathway in plants showing metabolites and genes analyzed in this study. Metabolites quantified in this study are highlighted. Proto IX, protoporphyrin IX; Mg-Proto IX, Mg-protoporphyrin IX; Mg-Proto IX ME, Mg-protoporphyrin IX monomethyl ester; Pchlide, protochlorophyllide. Genes and enzymes that correspond to the gene names: *HEMA*, glutamyl-tRNA reductase; *GSA*, glutamate 1-semialdehyde aminotransferase; *ALAD*, ALA dehydratase; *PPO*, protoporphyrinogen oxidase; *FC2*, Fe-chelatase 2; *HO*, heme oxygenase; *CHLD*, d-subunit of Mg-chelatase; *CHLH*, H-subunit of Mg-chelatase; *CHLI*, I-subunit of Mg-chelatase; *PORB*, protochlorophyllide oxidoreductase B. Solid and dashed arrows indicate a single step and multiple steps, respectively.

Tetrapyrroles play an essential role in photosynthesis, cellular respiration, catalytic activities of enzymes, and signal transduction [1,2]. In higher plants, 5-aminolevulinic acid (ALA) is formed from glutamic acid and subsequently converted to protoporphyrin IX (Proto IX) through a variety of reactions [1] (Figure 1). Proto IX is the point at which the chlorophyll (Mg-porphyrin) and the heme (Fe-porphyrin) pathways diverge. Metabolites in the porphyrin biosynthetic pathway are highly photoreactive and can easily be excited and transfer the energy to O_2_. Proto IX and protochlorophyllide (Pchlide) are especially reactive with O_2_ to form reactive oxygen species (ROS), which is harmful to cells and causes the peroxidation of membrane lipids [3,4].

Porphyrin-containing compounds are cofactors of apoproteins involved in photosynthesis and electron transfer reactions [5]. Plants have a remarkable ability to adapt their photosynthetic machinery to environmental condition such as different light qualities. Acclimatory responses of plants to light quantity and light spectral distribution often involve changes in the relative amounts of pigment-binding protein complexes, such as light-harvesting chlorophyll proteins (Lhcs), accompanied by corresponding changes in chlorophyll content in chloroplasts [6,7]. This ensures the coordinated assembly of the photosynthetic apparatus and avoids cellular photosensitization by free photoreactive tetrapyrroles [8]. In addition, nuclear gene expression including *Lhc* is regulated by the functional and developmental states of chloroplasts via retrograde signaling [9].

Light is an important environmental factor affecting plant development and growth by regulating morphological changes [10,11] as well as possible metabolic pathways. In photobiological studies, light-emitting diodes (LEDs) are an efficient system of narrow-band light output that can change the wavelength of light in enclosed environment. Although a number of studies using LED lights have been performed on the effect of the light spectral quality on the plant growth and morphogenesis [11,12], the underlying mechanisms of the effect of different light spectra on porphyrin metabolism and retrograde signaling have not yet been elucidated.

In this study, we examined the effect of light quality on the regulation of plant growth and morphology in rice seedlings grown under different wavelengths of light. We attempted to control light quality for plant growth by using various wavelengths from LEDs (Supplemental Table S1). Moreover, to shed light on plant growth driven by a specific wavelength of light, we monitored the regulatory mechanism of porphyrin metabolites and expression levels of their biosynthetic genes. We also investigated how the expression of nuclear-encoded photosynthetic genes is regulated by different wavelength of LEDs. This study demonstrates that light qualities influence not only the growth properties of rice seedlings following different LED treatments through altering porphyrin metabolism, but also possibly retrograde signaling.

## 2. Results and Discussion

### 2.1. Effects of Different Wavelengths of LEDs on the Growth and Morphological Appearances of Rice Seedlings

**Figure 2:**
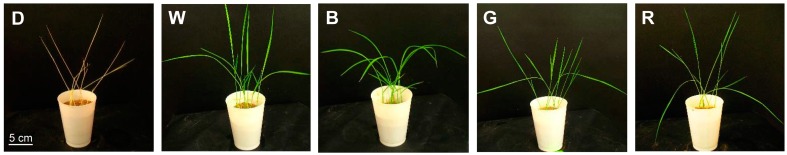
Effects of different wavelengths of LEDs on the growth parameters of rice seedlings. The five-day-old etiolated rice seedlings were exposed to different light-emitting diodes (LEDs) with a 14-h-light/10-h-dark photoperiod or under constant dark condition for 10 days. **D**, dark; **W**, broad spectrum-white LED (420−680 nm) as a control; **B**, blue LED (460−490 nm); **G**, green LED (520−550 nm); **R**, red LED (620−650 nm).

The effects of light quality on growth characteristics in rice seedlings were examined using different light wavelengths from LEDs. The five-day-old etiolated rice seedlings were exposed to white, blue, green, or red LED light in cycles of 14 h light and 10 h dark or maintained in continuous darkness for either 5 or 10 days. Exposure of etiolated seedlings to different wavelengths not only greatly enhanced plant growth, but also altered morphological characteristics (Figure 2). In response to five days of LED illumination, shoot height of seedling treated with white or blue LED were shorter than those of green LED, red LED, or dark conditions (Table 1). Leaf area of blue LED, as indicated by leaf length and width, was the greatest among all LED treatments, whereas dry biomass did not prominently differ.

Following prolonged exposure to 10 days of various LED light, the shoot height of rice seedlings treated with white LED or red LED was taller than that of blue LED, green LED or dark condition (Table 1). Rice seedlings grown in darkness initially showed a faster growth, but their growth was subdued in the subsequent phase, which was probably due to depletion of nutrition in the endosperms. By contrast, seedlings grown under white LED had a faster developmental pace in the later phase of the light exposure, implying that photosynthetic production is crucial for robust growth in this phase. The blue light signal inhibits elongation of leaf sheaths through repression of gibberellin (GA) biosynthetic-related genes and induction of GA inactivation-related genes [13]. Short shoot height of rice seedlings under blue LED might be connected with the report. Leaf length of rice seedlings treated with green LED was significantly shorter than seedlings treated with other LEDs, with the longest leaf length in seedlings with white LED. Noticeably, blue LED-illuminated seedlings showed a significant increase in leaf width compared to white LED. Unrolling (leaf width) of leaves was also promoted by blue light in etiolated rice seedlings, but not by red light [14]. Leaf width of orchid seedlings was expanded under white and blue LEDs and was narrower under green and red LEDs [15]. The seedlings treated with white or blue LEDs produced significantly greater dry biomass than other LEDs (Table 1). Wheat grown under red LED + 10% blue fluorescent light had greater shoot dry matter accumulation relative to wheat grown under red LED [16]. In higher plants, blue light is mainly perceived by cryptochromes and phototropins, which subsequently mediate a variety of blue light-dependent responses including phototropism, photomorphogenesis, stomatal opening, and rapid inhibition of hypocotyl elongation [12,17]. In our study, leaf growth and shoot biomass were greatly promoted by white and blue light, demonstrating that morphology and growth of rice seedlings were controlled by the light quality of LEDs.

### 2.2. Effects of Different Wavelengths of LEDs on the Levels of Photosynthetic Pigments and Porphyrin Metabolites

**Figure 3:**
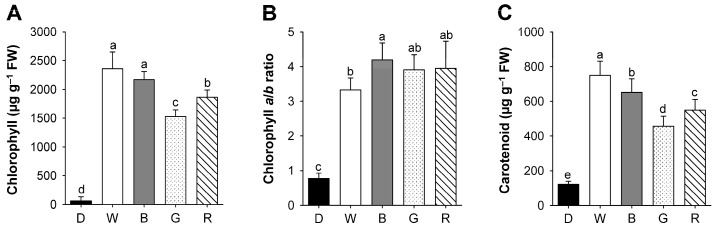
Effects of different wavelengths of LEDs on the content of total chlorophyll (**A**); chlorophyll *a*/*b* ratio (**B**); and carotenoid (**C**) in rice seedlings. The plants were subjected to the same treatments as in Figure 2, and treatment notations are the same as in Figure 2. The data represent the mean ± SD (*n* = 6). Means denoted by the same letter did not differ significantly at *p* < 0.05 according to Duncan’s multiple range test.

To assess the effect of different light qualities on photosynthetic pigments, we quantitated the amount of total chlorophylls and carotenoids in rice seedlings grown under various LED lights having different wavelengths. The seedlings grown in constant dark conditions showed a negligible level of chlorophylls, but rapidly accumulated chlorophylls in response to LED illumination (Figure 3). There were considerable differences in the content of chlorophyll, which is the end product of porphyrin biosynthetic pathway, when rice seedlings were grown under different LED sources. In comparison to broad-spectrum white LED, chlorophyll contents of green and red LEDs were decreased by approximately 35% and 21%, respectively, whereas chlorophyll contents in blue LED were similar to those of white LED (Figure 3A). In etiolated pea seedlings, illumination with blue light caused a large increment of chlorophyll within 48 h, while white light induced a small increment [18]. On the other hand, the chlorophyll *a*/*b* ratio increased in blue, red, and green LEDs compared to that of white LED, with the greatest increase in blue LED (Figure 3B). Elevated chlorophyll *a*/*b* ratio, particularly in blue LED, could reflect a reduced light-harvesting antenna size of photosystem II (PSII). Levels of total carotenoids also exhibited declines in blue, green, and red LEDs, compared to white LED (Figure 3C). These results indicate that a narrow spectrum LED influenced the chlorophyll *a*/*b* ratio as well as content of chlorophylls and carotenoids. Particularly, rice seedlings grown under blue LED sustained higher levels of chlorophylls and carotenoids than those of green and red LEDs. Blue light is important for chloroplast development and chlorophyll formation [18,19].

**Figure 4:**
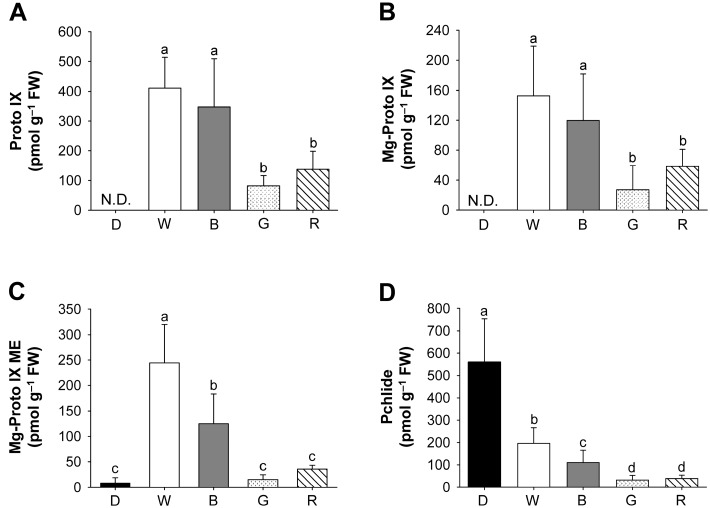
Effects of different wavelengths of LEDs on the distribution of porphyrin intermediates. (**A**) Proto IX; (**B**) Mg-Proto IX; (**C**) Mg-Proto IX ME; and (**D**) Pchlide. The plants were subjected to the same treatments as in Figure 2, and treatment notations are the same as in Figure 2. N.D., not detected. The data represent the mean ± SD (*n* = 6–8). Means denoted by the same letter did not differ significantly at *p* < 0.05 according to Duncan’s multiple range test.

To explore the relationship between porphyrin biosynthesis and light quality, we examined the effect of different LEDs on porphyrin intermediates of the biosynthetic pathway in rice seedlings. Although negligible amount of Mg-Proto IX monomethyl ester (ME) and great accumulation of Pchlide were observed under dark conditions, Proto IX and Mg-Proto IX were not detected (Figure 4). Levels of Proto IX and Mg-Proto IX in seedlings treated with blue LED were similar to those of white LED, whereas levels of Mg-Proto IX ME and Pchlide were decreased by 49% and 44%, respectively (Figure 4). Both green and red LED treatments greatly decreased levels of Proto IX and Mg-tetrapyrroles including Mg-Proto IX, Mg-Proto IX ME, and Pchlide, with lower levels in green LED. Our data provide evidence that a narrow spectrum of each LED treatment has a striking effect on the porphyrin metabolism in rice seedlings. Particularly, blue light appears to be essential for efficient biosynthesis of Proto IX and Mg-porphyrins. Metabolic control in the porphyrin biosynthetic pathway is critical for the fitness of plants [20]. Therefore, plants can trigger severe photodynamic damage if this control mechanism is perturbed by altered growth conditions [21,22].

### 2.3. Different Wavelengths of LEDs Influence Porphyrin Biosynthesis and Retrograde Signaling

**Figure 5:**
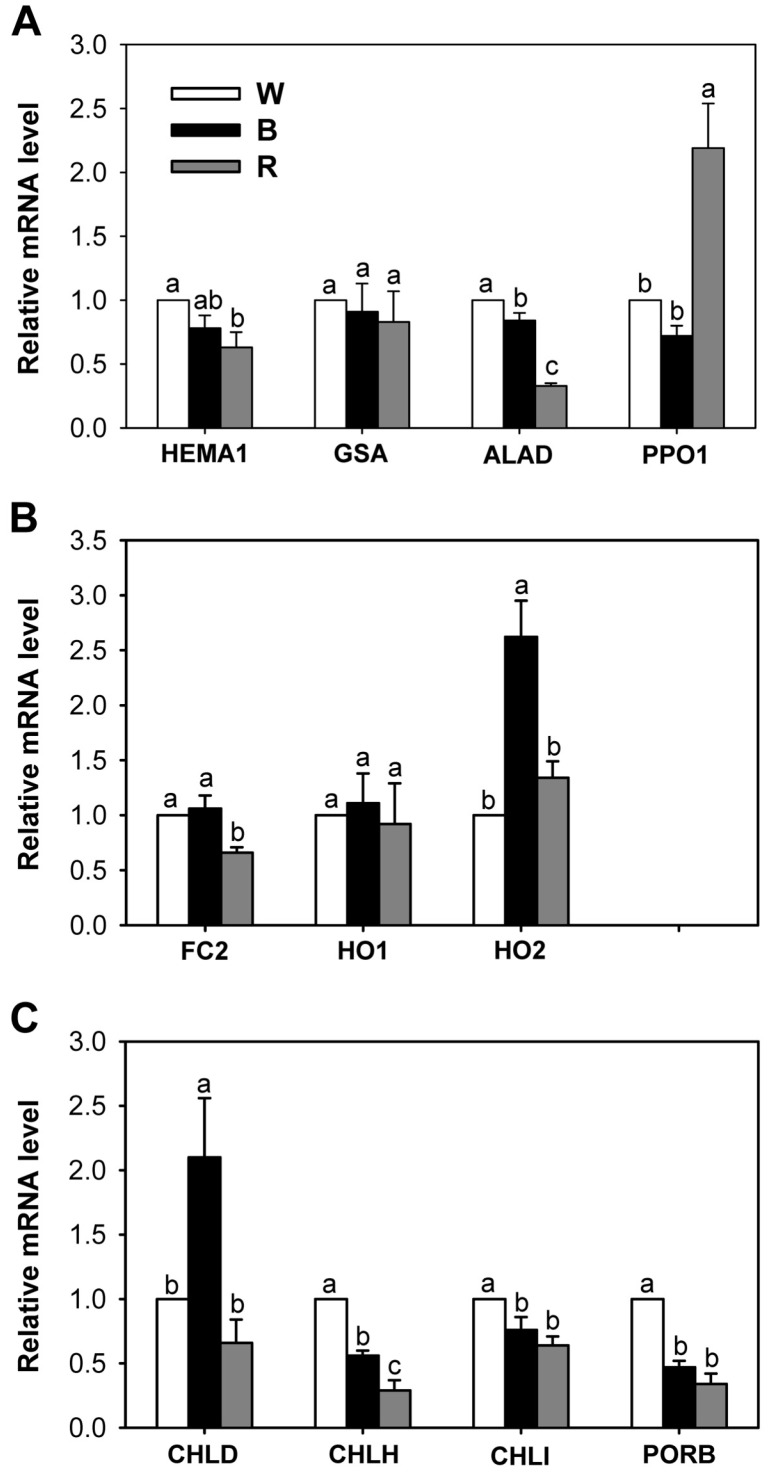
Effects of different wavelengths of LEDs on the expression of genes encoding the porphyrin pathway enzymes. (**A**) Common branch; (**B**) Fe-porphyrin branch; and (**C**) Mg-porphyrin branch. The plants were subjected to the same treatments as in Figure 2. Treatment notations are the same as in Figure 2. Total RNAs were purified from plants and reverse transcribed. The resultant cDNAs were used as templates for qRT-PCR using *Actin* as an internal control. The white LED (control) was used for normalization, with the expression level of the sample set to 1. Error bars indicate SEM from three independent experiments (*n* = 3); each experiment has three biological replicates. Means denoted by the same letter did not differ significantly at *p* < 0.05 according to Duncan’s multiple range test.

Photo-excitation of porphyrins with blue light, particularly in the wavelength range of 405–470 nm, results in the production of ROS, such as ^1^O_2_, H_2_O_2_, and O^2–^ [23]. Therefore, we focused on the effects of blue and red light, both of which are sensed by specific photoreceptors in plants [24,25]. Expression levels of biosynthetic genes in the porphyrin pathway were examined using qRT-PCR in rice seedlings grown under broad spectrum-white LED as well as blue and red LEDs. During prolonged red and blue LED treatments, transcript levels of *HEMA1* involving ALA-synthesizing activity and *ALAD*—which encodes ALA dehydratase—were lower than those of white LED (Figure 5A), implying that a specific wavelength of light affects the early step of porphyrin biosynthesis.

Transcript levels of *Glutamate-1-Semialdehyde Aminotransferase* (*GSA*), which also involves ALA-synthesizing activity, were similar among all three LEDs. Transcript levels of *PPO1* encoding the protoporphyrinogen oxidase (PPO), which produces Proto IX in the last step before the branch point, were greatly upregulated in seedlings treated with red LED, compared to those of white and blue LEDs. However, this upregulation of *PPO1* in red LED did not result in a corresponding increase in Proto IX.

As different wavelengths of LEDs affected the levels of Mg-porphyrin intermediates and chlorophylls, we investigated whether these changes can be explained by a regulatory switch in Proto IX distribution through regulating the genes encoding enzymes in Fe- and Mg-porphyrin branches. The transcript level of *FC2*—which encodes the plastidic isoform of Fe-chelatase—in seedlings treated with blue LED was similar to that of white LED, but decreased in red LED (Figure 5B). The transcript levels of *HO1* encoding heme oxygenase (HO)—which catalyzes the oxygen-dependent degradation of heme to biliverdin IXα [26,27]—were the same in all LED treatments. Interestingly, seedlings treated with blue LED exhibited a 2.6-fold increase in transcript level of *HO2*, compared to seedlings treated with white LED, implying more conversion of heme to biliverdin IXα. The principal HO reaction product, biliverdin**-**IXα, prevents oxidative cell damage as part of the antioxidant machinery in animal cells [28]. Its role in protection against oxidative stress in plant systems is also suggested by other reports [29,30,31]. In addition, HO2 shows strong binding of the heme precursor Proto IX in vitro, implicating a possible function of HO2 in the regulation of tetrapyrrole metabolism [32]. The high transcript level of *HO2* in seedlings treated with blue LED might contribute to scavenging the photosensitizing effect of Proto IX.

Among the genes encoding the three Mg-chelatase subunits CHLD, CHLH, and CHLI in the Mg-porphyrin branch, transcript levels of *CHLH* and *CHLI* decreased in seedlings treated with blue or red LED compared to white LED, whereas transcript level of *CHLD* in blue LED exhibited a two-fold increase (Figure 5C). CHLH is thought to have an additional function in mediating plastid-to-nucleus retrograde signaling by controlling the metabolism of Mg-Proto IX, which is an important molecule for retrograde signaling [33,34]. Both blue and red LEDs resulted in lower transcript levels of *Protochlorophyllide Oxidoreductase* (*PORB*), encoding the enzyme that generates chlorophyllide from Pchlide, than white LED, while chlorophyll contents did not significantly differ in samples taken from plants exposed to white and blue LEDs (Figure 3). This discrepancy is referred to as differential post**-**transcriptional controls under different LEDs. Notably, upregulations of *HO2* and *CHLD* under blue LED as well as *PPO1* under red LED suggest their important roles as key regulatory genes in porphyrin metabolism under altered light environment. Our results demonstrate that light spectral quality, as indicated by different wavelengths, controls levels of porphyrin metabolites through regulating expression of genes involved in porphyrin biosynthesis pathway.

**Figure 6:**
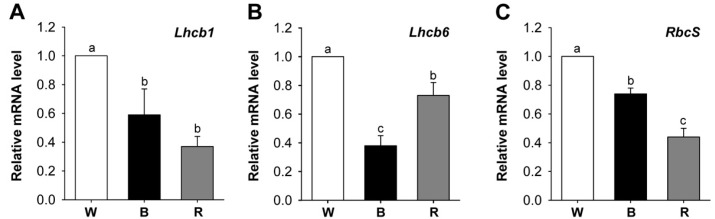
Effects of different wavelengths of LEDs on the expression of nuclear-encoded photosynthetic genes encoding the chlorophyll-binding proteins. (**A**) *Lhcb1*; (**B**) *Lhcb2*; and (**C**) *RbcS*. The plants were subjected to the same treatments as in Figure 2. Treatment notations are the same as in Figure 2. Total RNAs were purified from plants and reverse transcribed. The resultant cDNAs were used as templates for qRT-PCR using *Actin* as an internal control. The white LED (control) was used for normalization, with the expression level of the sample set to 1. Error bars indicate SEM from three independent experiments (*n* = 3); each experiment has three biological replicates. Means denoted by the same letter did not differ significantly at *p* < 0.05 according to Duncan’s multiple range test.

Expression of *Lhc* genes depends on the developmental and functional state of chloroplasts [35]. Transcript levels of *Lhcb*, the genes for Lhcs of PSII, and the *RbcS* gene encoding the small subunit of Rubisco were determined as markers of photosynthetic gene expression patterns in rice seedlings grown under different LEDs. The expression levels of nuclear-encoded photosynthetic genes—including *Lhcb1*, *Lhcb6*, and *RbcS*—decreased after exposure to blue and red LEDs, compared to those of white LED (Figure 6). The greater declines in transcript levels of *Lhcb* and *RbcS* than in chlorophyll content can be explained by the reduced light-harvesting antenna size of PSII, as indicated by the increased chlorophyll *a*/*b* ratio in blue and red LEDs (Figure 3 and Figure 6). It might be expected that the regulatory patterns of *CHLH*, *CHLI*, and *PORB* in Mg-porphyrin branch as well as *Lhcb* and *RbcS* by white light would correspond to the combined effects of red and blue light. Signals from the chloroplast regulate the expression of the nuclear-encoded genes, *Lhcb* [34] and *HEMA1*, which encodes glutamyl tRNA reductase, the first committed step of tetrapyrrole biosynthesis in plants [2,36,37]. Particular porphyrin molecules may absorb a specific wavelength of light, thereby involving retrograde signaling which sends signals to the nucleus to control expression of nuclear-encoded photosynthetic genes encoding the pigment-binding protein complex of the photosynthetic machinery.

## 3. Materials and Methods

### 3.1. Plant Growth and Light Treatment Conditions

Rice (*Oryza sativa* cv. Dongjin) seeds were germinated at 25 °C under dark condition for five days before irradiation. Then, the rice seedlings were grown under various types of light-emitting diodes (LEDs; Union LED Electronic Co., Siheung-si, Korea) for 10 days. In each cycle, about 100 seedlings were used for each LED treatment. Each LED treatment was conducted in separately controlled chambers to be free from spectral interference among treatments. The LED array chambers were programmed to provide a 14-h-light/10-h-dark photoperiod at photosynthetic photon flux maintained of approximately 150 μmol·m^−2^·s^−1^. The rice seedlings were grown under four different light sources (Supplemental Table S1) with broad spectrum-white LED (420−680 nm) as a control, blue LED (460−490 nm), green LED (520−550 nm), and red LED (620−650 nm), or under constant dark conditions. For pigment and RNA samples, shoot parts of rice seedlings were sampled at 4 h after illumination (11 AM) on the last day of LED treatments.

### 3.2. Determination of Morphological Characteristics

For measurement of shoot height, the length from seed (endosperm) to the top of the leaf in rice seedlings was measured by a Vernier Caliper. The length and the width of the youngest, fully developed leaf in each seedling were measured for the estimation of leaf area. To measure dry biomass, the shoot part of rice seedlings were dried at 80 °C for 48 h and weighed.

### 3.3. Extraction and Analysis of Porphyrin Intermediates and Photosynthetic Pigments

For measurement of porphyrin content, porphyrins were extracted and analyzed following the method of Lermontova and Grimm [38] with some modifications. Leaf tissue (0.1 g) from rice seedlings after 10 days of light exposures or darkness were macerated with liquid N_2_ and extracted in 1.5 mL of extraction buffer (methanol:acetone:0.1 N NaOH, 9:10:1, *v*/*v*/*v*) [31]. The homogenate was centrifuged at 10,000× *g* for 10 min and the supernatant was taken for HPLC analysis. Porphyrin intermediates were separated by HPLC (1525 Binary HPLC Pump, Waters, Milford, MA, USA) using a Novapak C_18_ column (4 μm particle size, 4.6 mm × 250 mm, Waters). Porphyrin intermediates were eluted with a solvent system of 0.1 M ammonium phosphate (pH 5.8) and methanol at a flow rate of 1 mL·min^–1^. The elution profile of porphyrins from column was monitored with a fluorescence detector (2474, Waters) at excitation and emission wavelengths of 400 and 630 nm for Proto IX, 440 and 630 nm for Pchlide, and 415 and 595 nm for Mg-Proto IX and Mg-Proto IX ME, respectively. All porphyrins were identified and quantified using authentic standards. The contents of total chlorophylls and carotenoids were spectrophotometrically determined according to the method of Lichtenthaler [39].

### 3.4. RNA Extraction and qRT-PCR Analysis

Total RNA was isolated from leaf tissue (0.1 g) after 10 days of light exposure using TRIZOL Reagent (Invitrogen, Carlsbad, CA, USA) in accordance with manufacturer’s instructions. The 5 µg of purified total RNA from each sample was used for the first strand cDNA synthesis by the reverse transcription reaction (SuperScript III First-Strand Synthesis System, Invitrogen) in the total volume of 20 µL following manufacturer’s instructions. Subsequently, 50 ng of cDNA and specific primers for genes (Supplemental Table S2) were used for qRT-PCR analysis. The qRT-PCR analysis was performed with the 7300 Real-Time PCR system (Applied Biosystems, Waltham, MA, USA) using Power SYBR Green PCR Master Mix (Applied Biosystems). The thermal cycling of qRT-PCR program consisted of an initial step at 50 °C for 2 min, at 95 °C for 10 min, and 40 cycles of 15 s at 95 °C, and 1 min at 60 °C [21]. A melting curve analysis was performed after every PCR reaction to confirm the accuracy of each amplified product. All reactions were set up in triplicate. The sample of white LED treatment was used as the calibrator, with the expression level of the sample set to 1. *Actin* was used as the internal control.

### 3.5. Data Analysis

Data were expressed as means ± SE. Differences were analyzed by the Duncan’s multiple range test. *p*-values < 0.05 were considered to be significant. The analyses were performed using the *SPSS* software (SPSS Inc., Chicago, IL, USA).

## 4. Conclusions

In this study, levels of porphyrin metabolites and photosynthetic pigments as well as growth properties were greatly influenced by different wavelengths of LEDs. The greater leaf width and dry biomass in rice seedlings grown under blue LED and broad spectrum-white LED may be related to the fact that porphyrin compounds absorb blue wavelength intensely. Our data also show that different wavelengths of LEDs have marked effects on the metabolic regulation of the porphyrin biosynthetic pathway. In comparison to white LED, most porphyrin genes in the Fe-porphyrin branch remained constant or upregulated under blue and red LEDs, while most genes in the Mg-porphyrin branch were downregulated. Particularly, the accumulation of *HO* under blue LED may be due to a higher energy level of blue light, however, the relevant mechanism is unknown. Expression levels of *Lhcb* and *RbcS* noticeably decreased after exposure to blue and red LEDs, compared to white LED, implying that particular wavelengths of light from different LEDs may possibly alter retrograde signaling which sends chloroplast signals to the nucleus to control expression of nuclear-encoded photosynthetic genes.

Further studies are needed to elucidate the signaling pathway involved in the light wavelength-dependent physiological modifications in plants. This may act through altering porphyrin biosynthesis, although it remains necessary to explain how a specific LED wavelength leads to the altered porphyrin biosynthesis.

## Figures and Tables

**Table 1:** Effect of LED lights on the morphological characteristics of rice seedlings after different wavelengths of LED illumination.

Treatment	Shoot Height (cm)	Leaf Length (cm)	Leaf Width (cm)	Dry Weight (mg·plant^−1^)
**5 days**				
Dark	17.9 ± 1.6 a	6.1 ± 1.0 b	0.19 ± 0.01 d	11.7 ± 1.3 ab
White	14.3 ± 1.1 c	5.4 ± 1.0 c	0.24 ± 0.03 b	11.6 ± 1.5 abc
Blue	13.6 ± 0.7 d	7.1 ± 0.9 a	0.33 ± 0.05 a	12.0 ± 1.4 a
Green	16.9 ± 1.3 b	5.8 ± 1.0 ab	0.22 ± 0.02 c	10.8 ± 1.9 c
Red	17.7 ± 1.3 a	6.3 ± 1.2 b	0.20 ± 0.02 d	11.1 ± 1.7 bc
**10 days**				
Dark	22.5 ± 3.3 c	7.4 ± 2.1 d	0.26 ± 0.03 d	10.6 ± 1.6 c
White	27.3 ± 3.2 a	17.0 ± 2.5 a	0.33 ± 0.03 b	15.7 ± 2.2 a
Blue	22.4 ± 2.3 c	13.8 ± 1.5 b	0.37 ± 0.02 a	14.9 ± 2.2 a
Green	24.4 ± 1.5 b	11.8 ± 3.0 c	0.32 ± 0.03 b	12.7 ± 2.2 b
Red	27.5 ± 1.7 a	14.1 ± 2.2 b	0.28 ± 0.03 c	12.6 ± 1.3 b

The five-day-old rice seedlings were exposed to different LEDs with a 14-h-light/10-h-dark photoperiod or under constant dark condition for either 5 or 10 days. The data represent the mean ± SD of at least 30 plants from two independent experiments. Within each column, means denoted by the same letter did not differ significantly at *p* < 0.05 according to Duncan’s multiple range test.

## Supplementary Materials

Supplementary materials can be found at www.mdpi.com/1422-0067/18/3/641/s1.
