# Supplementary material for: Effects of Light-Emitting Diode Irradiation on Growth Characteristics and Regulation of Porphyrin Biosynthesis in Rice Seedlings
Source: Int J Mol Sci. 2017 Mar 16;18(3):641. doi: 10.3390/ijms18030641 (PMC5372653; doi:10.3390/ijms18030641)
Supplement: Supplementary file 1 [file ijms-18-00641-s001.pdf]

**Table S1.** Emission spectra of LEDs used in this study.

| LED | Wavelength range (nm) |
|---|---|
| White | 420–680 nm |
| Blue | 460–490 nm |
| Green | 520–550 nm |
| Red | 620–650 nm |

**Table S2.** Primers used for qRT-PCR assays.

| Gene | Primer sequence |
|---|---|
| *HEMA1* | F: GCTATGGGTGGTGTTCGACT |
| | R: CGATCTTCTGGAGGCACTTC |
| *GSA* | F: CTCCGTGACTTGACGAAACA |
| | R: GTAGGTTCCAGGCTCCATCA |
| *ALAD* | F: GTCCACCGTCTCCTTCTCC |
| | R: TGTCAAGTCAAGAGGCCTGA |
| *PPO1* | F: ACAGTTCCTCATTGGCCATC |
| | R: CCCATGAAATTTTTGCTGCT |
| *CHLD* | F: TGGGACAGCAAAGACAGTGA |
| | R: AAGGCCAGGTTGAAACACAG |
| *CHLH* | F: GTGTGGGTTGCGTTCTTTTT |
| | R: GGTGACAATGTGGCTCCTCT |
| *CHLI* | F: TGTGCTTCTGGATTCTGCTG |
| | R: GCTGGAGCTTGTCTTGTTCC |
| *PORB* | F: GTGAATTGCCAGGTTTTCGT |
| | R: GCAATTAGCAAAGCTGCACA |
| *FC2* | F: TTGGTGCTATGGCAGTTTCA |
| | R: AGTGGAACAAAGGCAGGATG |
| *HO1* | F: AGCGCTAGCAGTAGCAGGAG |
| | R: GCTCCTTCTCCCCTTCCTT |
| *HO2* | F: AGGGACCTAGCAGCCCTAAC |
| | R: CCCGTATCGTCCATCTTGAG |
| *Lhcb1* | F: CAGCTCTCACAGCTCACTGC |
| | R: GTGTCCCACCCGTAGTCG |
| *Lhcb6* | F: CTCATGGGCTGGGTAGAGTC |
| | R: CGGCCTCGAAGTAGAAGATG |

| | |
|---|---|
| *RbcS* | F: GCAGCTTTGTTTTGGGCTAT |
| | R: CCGGAGCTTTGTTTTCACAT |
| *Actin* | F: CTTCATAGGAATGGAAGCTGCGGGTA |
| | R: CGACCACCTTGATCTTCATGCTGCTA |

F, forward; R, reverse.
